# Ectopic Gallstone Presenting As Small Bowel Obstruction: A Diagnostic Challenge

**DOI:** 10.7759/cureus.110495

**Published:** 2026-06-08

**Authors:** Deneisy H Trinidad Cruz, Hector S Jasso Rodriguez, Jose M Zermeño, Gloria J Coello Uribe

**Affiliations:** 1 Surgery, Hospital General Instituto de Seguridad y Servicios Sociales de los Trabajadores del Estado (ISSSTE) Aguascalientes, Aguascalientes, MEX

**Keywords:** bowel obstruction, cholecystoduodenal fistula, enterolithotomy, gallstone ileus, pneumobilia

## Abstract

Gallstone ileus represents an infrequent etiology of mechanical bowel obstruction and may be difficult to identify because of its nonspecific clinical manifestations. We describe the case of an 80-year-old woman who presented with abdominal pain, nausea, vomiting, and constipation. Computed tomography demonstrated pneumobilia, a cholecystoduodenal fistula, and an ectopic gallstone lodged at the ileocecal valve. Surgical exploration confirmed the diagnosis, and the patient underwent enterotomy with stone extraction, partial cholecystectomy, and fistula repair. Recovery after surgery was favorable and without major complications. This report highlights the importance of considering gallstone ileus in older adults with bowel obstruction and emphasizes the usefulness of computed tomography for timely diagnosis and surgical planning. Management should be individualized according to intraoperative findings and the patient’s overall clinical status.

## Introduction

Gallstone ileus is an uncommon complication of cholelithiasis caused by the migration of one or more gallstones into the gastrointestinal tract through a biliary-enteric fistula, resulting in mechanical intestinal obstruction. Historical descriptions of this entity date back to the 17th century, while larger clinical series were later reported by Zaliekas and Munson [1]. Despite its rarity, gallstone ileus remains an important diagnostic consideration in elderly patients presenting with symptoms of bowel obstruction [2].

Clinical manifestations are frequently nonspecific and may include abdominal pain, nausea, vomiting, abdominal distension, and constipation, which can delay diagnosis [3]. Imaging studies, particularly computed tomography, play a central role in identifying characteristic findings such as pneumobilia, ectopic gallstones, and intestinal obstruction [4]. Surgical treatment remains the cornerstone of management, although the optimal approach should be tailored to the patient’s clinical condition and intraoperative findings [5].

## Case presentation

An 80-year-old woman with a history of diabetes mellitus, poorly controlled hypertension, colpoperineoplasty, and right nephrectomy arrived with a three-day history of abdominal pain, constipation, nausea, and fever. She had initially been managed by a private physician but showed no improvement. Over the following 24 hours, her symptoms worsened, prompting hospital admission.

On arrival, she exhibited signs of bowel obstruction, including severe abdominal pain localized to the epigastrium and right upper quadrant, bilious vomiting, and obstipation. Laboratory evaluation demonstrated leukocytosis (13.4 ×10³/µL) with neutrophilia (82%), as well as elevated hepatocellular and cholestatic biochemical markers, including alanine aminotransferase, aspartate aminotransferase, alkaline phosphatase, and gamma-glutamyl transferase (Table 1).

**Table 1:** Biochemical tests Liver function test and complete blood count.

Parameter	Value	Reference Range
Total bilirubin	1.43 mg/dL	0.2-1.3 mg/dL
Conjugated bilirubin	0.64 mg/dL	0-0.3 mg/dL
Unconjugated bilirubin	0.79 mg/dL	0-1.0 mg/dL
Alanine aminotransferase (ALT)	131 U/L	9-72 U/L
Aspartate aminotransferase (AST)	112 U/L	14-59 U/L
Lactate dehydrogenase (LDH)	179 U/L	120-246 U/L
Alkaline phosphatase (ALP)	368 U/L	44-147 U/L
Gamma-glutamyl transferase (GGT)	379 U/L	15-73 U/L
Albumin	3.6 g/dL	3.5-5 g/dL
Amylase	76 U/L	40-140 U/L
Lipase	110 U/L	10-120 U/L
Leukocytes	13.4 ×10^3^/µL	4.6-10.2 ×10^3^/µL
Hematocrit	45.6%	37.7%-53.7%
Hemoglobin	15.6 g/dL	12.2-18.1 g/dL
Platelets	162 ×10^3^/µL	142-424 ×10^3^/µL
Neutrophils	82%	37%-80%

Abdominal CT scan showed pneumobilia and a cholecystoduodenal fistula (Figures 1A, 1B), as well as dilated bowel loops and a hyperdense lesion at the ileocecal valve consistent with an ectopic gallstone (Figure 2).

**Figure 1:**
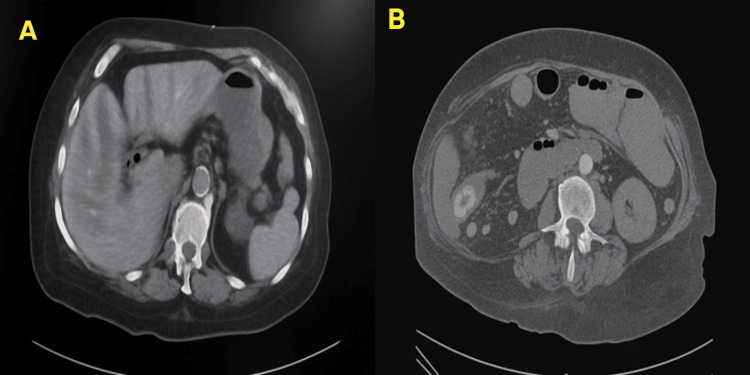
Non-contrast abdominal computed tomography: axial view (A) Axial view demonstrating pneumobilia (small air bubbles or hypodense foci within the intrahepatic biliary tree). (B) More caudal view of A, showing dilated bowel loops, along with a hyperdense image corresponding to an ectopic gallstone at the ileocecal valve.

**Figure 2:**
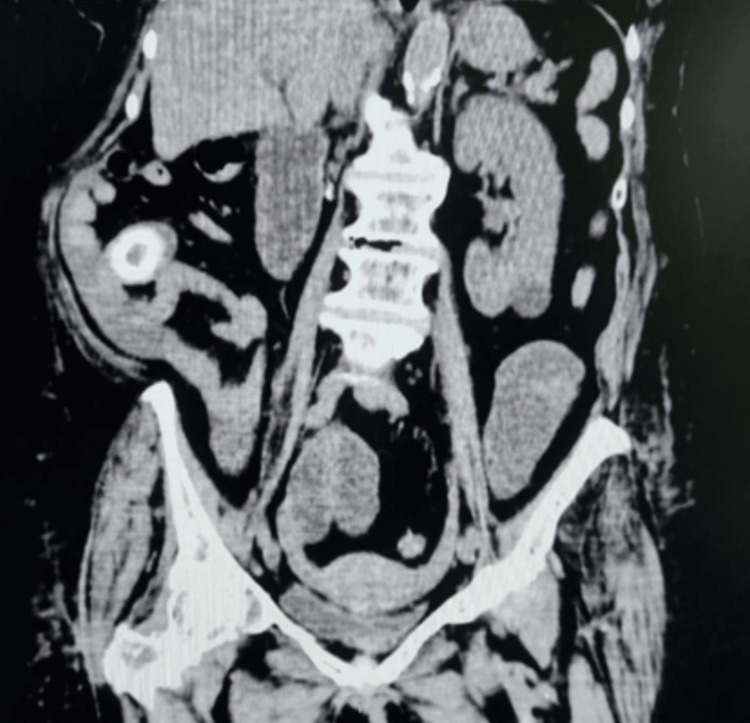
Non-contrast abdominal computed tomography: coronal view Coronal view demonstrating an ectopic gallstone within the intestinal lumen.

The patient underwent exploratory laparotomy, which confirmed the presence of a cholecystoduodenal fistula without bile leakage and a single 32×30 mm gallstone impacted in the terminal ileum. Partial cholecystectomy, fistulectomy, and enterotomy were then performed. The patient had an uneventful post-operative recovery and was discharged in stable condition.

## Discussion

Gallstone ileus is an uncommon cause of mechanical bowel obstruction that predominantly affects elderly women with multiple comorbidities, such as diabetes mellitus and hypertension. In the present case, the patient was an 80-year-old female, which is consistent with the typical demographic described in the literature, where the average age ranges between 70 and 80 years, and there is a clear female predominance [2,4,6]. 

Advanced age and associated conditions such as diabetes mellitus are recognized predisposing factors. Diabetes has been linked to gallstone formation through mechanisms including impaired gallbladder motility, bile stasis, and altered lipid metabolism, all of which contribute to cholesterol supersaturation and lithogenesis [2,5,7]. These factors contribute to the development of gallstone disease and its complications. This condition develops secondary to the formation of a biliary-enteric fistula, most commonly cholecystoduodenal, which allows the passage of gallstones into the gastrointestinal tract, as observed in our patient [2-4].

The terminal ileum is the most common site of gallstone impaction due to its narrower lumen and reduced peristalsis. In our case, the obstructing gallstone was located at the distal ileum, consistent with this well-described pattern. Furthermore, gallstones larger than 2-2.5 cm are more likely to cause obstruction, whereas smaller stones usually pass spontaneously through the gastrointestinal tract [2,4]. Clinically, gallstone ileus presents with nonspecific symptoms of small bowel obstruction, including abdominal pain, nausea, vomiting, and obstipation, often leading to delays in diagnosis [2-4]. Notably, our patient did not present with jaundice, which is consistent with the literature reporting its presence in fewer than 15% of cases [3].

Laboratory findings in gallstone ileus are often nonspecific and may reflect the underlying inflammatory process rather than the severity of the obstruction itself. In this case, leukocytosis with neutrophilia was observed, consistent with an inflammatory response. Additionally, the patient demonstrated elevated hepatocellular and cholestatic biochemical markers, including aminotransferases, alkaline phosphatase, and gamma-glutamyl transferase, with only a mild elevation in total bilirubin. These findings may be related to the underlying biliary disease and the presence of a cholecystoenteric fistula. As reported in previous studies, laboratory abnormalities in gallstone ileus are variable and should be interpreted in conjunction with the clinical presentation and imaging findings, as neither normal nor abnormal liver biochemical tests are sufficiently sensitive or specific to establish or exclude the diagnosis [2,5].

Computed tomography (CT) is considered the diagnostic modality of choice for gallstone ileus [5,6,8]. In our patient, CT imaging demonstrated Rigler’s triad, consisting of pneumobilia, dilated bowel loops, and an ectopic gallstone distal to the site of obstruction, which is regarded as pathognomonic for this condition [6,8]. However, the complete triad is not always present, highlighting the importance of clinical suspicion [1,2].

Management of gallstone ileus remains controversial and should be individualized according to patient characteristics and clinical presentation. While acute cases with complete obstruction require prompt surgical intervention, alternative strategies such as staged procedures or minimally invasive approaches may be considered in selected patients [2,9,10]. The choice between enterolithotomy alone and a one-stage procedure including cholecystectomy and fistula repair remains debated [2,4,5].

Gallstone ileus is associated with a mortality rate ranging from 12% to 27% and morbidity approaching 50%, largely due to advanced age, comorbidities, and delays in diagnosis and treatment [3,4]. Therefore, early recognition and timely intervention are critical to improving patient outcomes. This case highlights the importance of maintaining a high index of suspicion in elderly patients presenting with bowel obstruction, even in the absence of jaundice or abnormal liver function tests. It also underscores the key role of CT imaging in establishing the diagnosis and supports an individualized surgical approach based on patient-specific factors [2,7,8].

## Conclusions

Gallstone ileus is a rare but clinically significant cause of mechanical bowel obstruction, particularly among elderly patients with a history of biliary disease. This case underscores the importance of considering gallstone ileus in the differential diagnosis of bowel obstruction, as clinical presentation and laboratory abnormalities may be variable. Computed tomography remains the diagnostic modality of choice, enabling identification of Rigler’s triad, accurate assessment of the size and location of the obstructing gallstone, and prompt surgical planning, all of which contribute to improved patient outcomes.

Although the optimal surgical approach remains controversial, current evidence supports an individualized, patient-centered surgical approach based on patient comorbidities, clinical status, and intraoperative findings. Comparative studies have demonstrated that enterolithotomy alone may be preferable in high-risk patients, while one-stage procedures can be considered in selected cases. Early diagnosis and tailored surgical management are essential to reduce morbidity and mortality associated with this condition and to improve overall patient outcomes.
